# Simultaneous atelectasis in human bocavirus infected monozygotic twins: was it plastic bronchitis?

**DOI:** 10.1186/1471-2431-13-209

**Published:** 2013-12-18

**Authors:** Christoph M Rüegger, Walter Bär, Peter Iseli

**Affiliations:** 1 Neonatal and Pediatric Intensive Care Unit, Graubuenden Cantonal Hospital, Chur, Switzerland; 2 Division of Neonatology, University Hospital Zurich, Frauenklinikstrasse 10, CH-8091 Zurich, Switzerland

**Keywords:** Bronchial casts, Plastic bronchitis, Atelectasis, Children, Respiratory tract infection, Human bocavirus

## Abstract

**Background:**

Plastic bronchitis is an extremely rare disease characterized by the formation of tracheobronchial airway casts, which are composed of a fibrinous exudate with rubber-like consistency and cause respiratory distress as a result of severe airflow obstruction. Bronchial casts may be associated with congenital and acquired cardiopathies, bronchopulmonary diseases leading to mucus hypersecretion, and pulmonary lymphatic abnormalities. In recent years, however, there is growing evidence that plastic bronchitis can also be triggered by common respiratory tract infections and thereby cause atelectasis even in otherwise healthy children.

**Case presentation:**

We report on 22-month-old monozygotic twins presenting with atelectasis triggered by a simple respiratory tract infection. The clinical, laboratory, and radiographic findings given, bronchial cast formation was suspected in both infants but could only be confirmed after bronchoscopy in the first case. Real-time polymerase chain reaction of the removed cast as well as nasal lavage fluid of both infants demonstrated strong positivity for human bocavirus.

**Conclusion:**

Our case report is the first to describe two simultaneously affected monozygotic twins and substantiates the hypothesis of a contributing genetic factor in the pathophysiology of this disease. In this second report related to human bocavirus, we show additional evidence that this condition can be triggered by a simple respiratory tract infection in previously healthy infants.

## Background

Plastic bronchitis is an extremely rare and unusual condition characterized by the formation of tenacious airway casts mimicking the three-dimensional architecture of the tracheobronchial tree [1]. This condition, which differs from ordinary mucus plugging by its cohesiveness, consistency, and typically difficult bronchoscopic removal [2], was first described in the early 19th century, but its pathophysiology is still unknown [3]. In a review of 42 cases of paediatric plastic bronchitis, Brogan et al. noted that 40% of affected patients had an underlying cardiac defect, 31% had asthma or allergic disease, and 29% had another or unknown disease. They found an overall mortality rate of 16%, reaching 28% for cardiac patients due to respiratory failure following central airway obstruction [1]. The most widely used classifications of plastic bronchitis were established by Seear et al. [4] based on the histology of the mucus plug and, more recently, by Madsen et al. [5], who divided plastic bronchitis into four etiological groups related to the associated conditions and cast histology (Table 1). The differential diagnosis encompasses different conditions with subtotal or total bronchial obstruction, such as lobar pneumonia, severe bronchial asthma, foreign body aspiration, and mucoid impaction.

**Table 1:** Classification schemes of plastic bronchitis

**Seear et al. 1997 (3)**	**Madsen et al. 2005 (4)**		
	**Associated disease**	**Histology**	**Pathophysiology**
**Type I (inflammatory) casts**	**Asthma and atopic diseases**	Fibrin with a dense eosinophilic infiltrate, Charcot-Leyden crystals	Hypersecretion of viscous mucus (dyscrasia)
- acute presentation			
**Type II (acellular) casts**	**Lymphatic disorders**	Chylous casts sometimes containing fibrin	Incompetence of lymphatic valves, mechanical disruption of the thoracic duct or one of its large tributaries, lymphangiectasia, lymphangiomatosis
- chronic or recurrent			
	**Structural congenital heart disease**	Acellular mucinous casts	High pulmonary venous pressure leading to an abnormal response of the bronchial epithelium resulting in excess mucus production
	**Sickle cell disease**	Fibrinous material composition and pigmented histiocytes in the surrounding fluid	Ischemia of the bronchial tree caused by vaso-occlusion leading to ciliary motility dysfunction

In recent years, however, there is growing evidence that plastic bronchitis can also be triggered by simple respiratory tract infections and thereby cause atelectasis even in otherwise healthy children [6,7]. In this article we describe two monozygotic twins without underlying conditions suffering from respiratory distress following a common, human bocavirus 1 (HBoV1) positive respiratory tract infection.

## Case presentation

### Case 1

**Figure 1:**
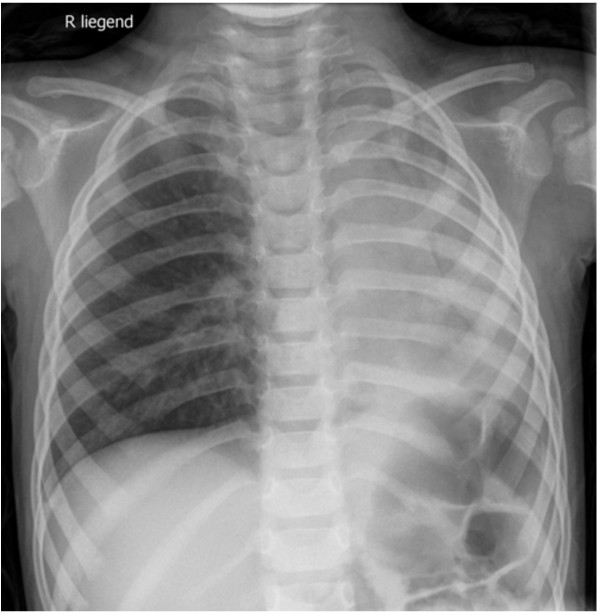
**Chest X-ray of case 1 taken on admission.** Abrupt termination of left main stem air shadow and collapse of left lung suggest complete obstruction of left bronchial tree.

**Figure 2:**
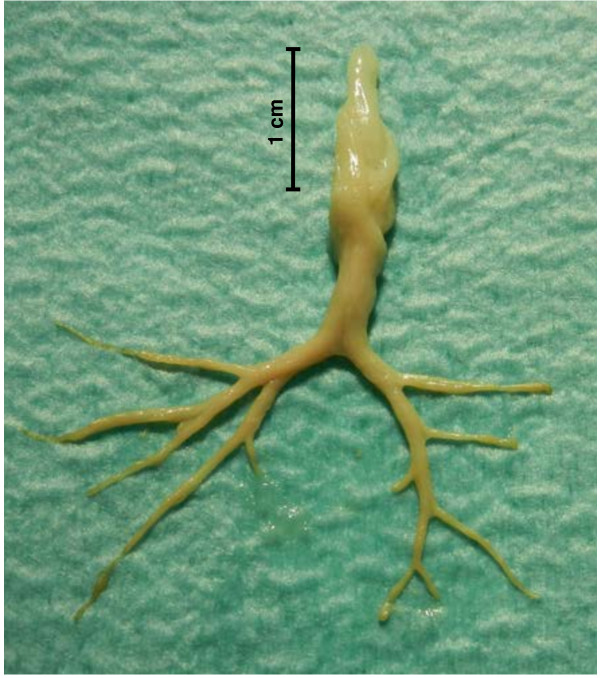
Bronchial cast removed from the left main stem bronchus, reproducing the bronchial segmentation of the left upper and lower lobes.

A 22-month-old boy presented with a three-day history of common cold and mild respiratory distress. Ambulant inhalation therapy with salbutamol was initiated, but the patient deteriorated. When admitted to the emergency room, his general condition was markedly reduced with signs of respiratory distress and decreased breath sounds over the left hemithorax (Figure 1). Rigid bronchoscopy was performed, and surprisingly, a complete tenacious bronchial cast was removed (Figure 2). Histopathology revealed a dense inflammatory infiltrate composed of fibrin, mucus, and eosinophils. Immediately after the intervention, ventilation was restored, and the clinical findings returned to nearly normal. Real-time polymerase chain reaction of both nasal lavage fluid and the bronchial cast demonstrated strong positivity for HBoV1. The patient was discharged after six days and is currently healthy.

### Case 2

**Figure 3:**
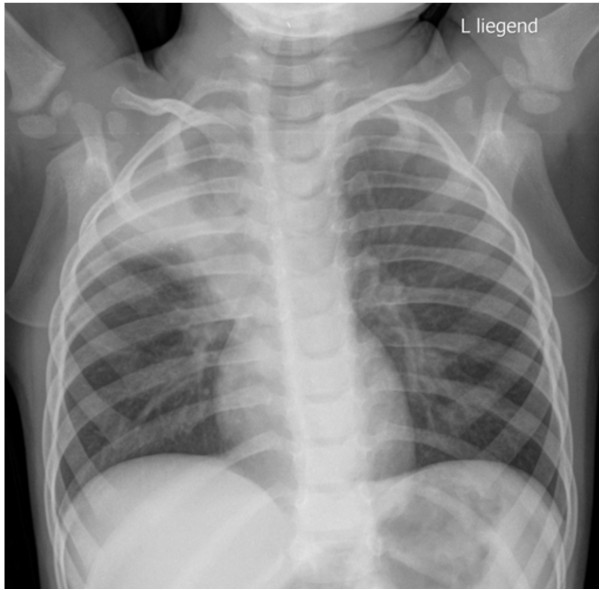
Chest X-ray of case 2 taken on admission with partial atelectasis of the right upper lobe with distinct signs of volume loss of the right lung.

The day following the admission of patient one, his monozygotic twin brother was referred to our emergency department because of worsening dyspnea and coughing after a one-week history of a common cold. A clinical examination found mild respiratory distress and decreased breath sounds over the right upper lung field (Figure 3). A nasopharyngeal aspirate was subjected to real-time polymerase chain reaction and was positive for HBoV1. As his general condition was only mildly affected, conservative therapy consisting of antibiotics (amoxicillin and clavulanic acid), inhaled corticosteroids and bronchodilators, and intensive respiratory physiotherapy was initiated. In the following days, ventilation of the right upper lung field ameliorated and returned to normal on the sixth day of hospitalization. The patient has not had any recurrences to date.

## Discussion

The current hypothesis regarding the pathogenesis of plastic bronchitis suggests that the final common pathway may be initiated by numerous stimuli and involves two requirements for cast formation, namely an underlying genetic predisposition and a second insult leading to the accumulation of mucin, fibrin, or chyle in the airways [5]. In our patients, the family history was unremarkable, and allergies, asthma, chronic lung diseases, and cardiac anomalies were absent. We can therefore only speculate about possible explanations for the excessive inflammatory response observed in these cases. Although a bocavirus infection simultaneously affecting monozygotic twins is an unusual event, acute bronchial obstruction due to simple respiratory tract infections is fairly common. Specifically, the observation several years ago that a significant number of infants with wheezing bronchitis had structures compatible with bronchial casts in their gastric fluid suggested that cast formation might be a common phenomenon in these children [8]. A combination of secretory hyperresponsiveness [9] and a severely disturbed mucociliary clearance system during viral infection [10,11] in the presence of an unrecognized predisposition appear to be the main drivers for plastic bronchitis in these cases. Interestingly, formations similar to virus-induced cast formations in children with influenza A (H1N1) [7,12] have been observed in chickens with avian influenza (H9N2) [13]. To the best of our knowledge, a case of human bocavirus-induced plastic bronchitis has previously been reported only once, in a 14-month-old, previously healthy patient [14]. HBoV1 was discovered in 2005 in nasopharyngeal secretions as a new member of the Parvoviridae [15]. It has since been recognized as the fourth most common cause of viral respiratory tract infections in children [16]. Recent studies demonstrated that HBoV1 efficiently infects the apical membrane of human airway epithelial cells, resulting in replication of progeny viruses and cytopathology [17]. Three additional human bocaviruses, HBoV2, -3 and -4, discovered in human stool samples, have since been characterized [18].

Because of the rarity of plastic bronchitis, therapy is not uniform and remains largely empiric based on clinical conditions. We believe that early and, if required, serial cast removal by rigid bronchoscopy is the mainstay of therapy and is potentially life-saving [5]. First-line adjunct therapies may include chest physiotherapy, airway humidification, and the application of aerosolized medication such as acetylcysteine [19] and DNAse [20] to improve mucociliary clearance. In patients with heart disease, optimization of cardiac output and, where appropriate, a low-fat diet or duct ligation is recommended [21]. Plastic bronchitis with type I inflammatory casts seems to be responsive to the use of anti-inflammatory therapeutics, including systemic or inhaled steroids [19]. In patients with recurrent episodes of plastic bronchitis, the administration of azithromycin [22] and macrolide antibiotics [23], as well as direct or inhaled administration of tissue-type plasminogen activator to the obstructing casts, [24] have been shown to resolve the episodes. Other fibrinolytic therapies such as heparin [25] and urokinase have been used with variable success.

In our cases, therapeutic strategies varied due to the different clinical presentations. In the first case, presenting with an inflammatory type I cast, a pragmatic approach of immediate rigid bronchoscopy was chosen due to the extent of atelectasis. Based on the rapid recovery after cast removal, our first-line follow-up treatment consisted of inhaled corticosteroids. However, the combination of acutely administered intravenous corticosteroids followed by inhaled corticosteroids has proven to be an effective and safe treatment of plastic bronchitis with type I inflammatory casts [26,27]. In plastic bronchitis caused by type II acellular casts, however, corticosteroids are often ineffective.

Given the rather mild clinical deterioration of case 2 with involvement of only one lobe, the same therapeutic work-up did not seem to be justified, and a conservative treatment with inhaled corticosteroids and bronchodilators was preferred. This regimen led to an overt improvement in course during the subsequent 6 days. Because no airway cast could be extracted and histologically examined, plastic bronchitis could not be confirmed according to the published diagnostic gold standard. However, several findings led to a strong suspicion of plastic bronchitis in case 2. The similar clinical symptoms, although milder in case 2 than in case 1, included a partial atelectasis of the right upper lobe with distinct signs of volume loss on X-ray. In addition, polymerase chain reaction of nasal lavage fluid was positive for HBoV1, as was the case in the patient’s twin brother. Last but not least, additional clinical and laboratory findings argued against a pneumonic process.

Because of the high risk of recurrent cast formation, the most critical component of plastic bronchitis management is close monitoring of any affected child, irrespective of the underlying condition, the initial extent and the course of the disease.

## Conclusion

The presented cases are the first to describe two simultaneously affected monozygotic twins and substantiate the hypothesis of a contributing genetic factor in the pathophysiology of this disease. In this second report related to HBoV1, we show additional evidence that this condition can be triggered by a simple respiratory tract infection in previously healthy infants. Different initial therapeutic strategies when facing a child with atelectasis and suspected plastic bronchitis include immediate bronchoscopy as well as mucolytic, anti-inflammatory, and fibrinolytic treatments depending on the underlying condition, the clinical and radiographic extent of the disease and the histopathologic type of airway cast.

### Consent

Written informed consent was obtained from the patient’s parents for publication of this case report and accompanying images.

## Competing interests

The authors declare that they have no competing interests.

## Authors’ contributions

CMR was responsible for literature review, conception and preparation of the manuscript. WB and PI participated in preparation and critical revision of the manuscript. All authors read and approved the final manuscript.

## Pre-publication history

The pre-publication history for this paper can be accessed here:

http://www.biomedcentral.com/1471-2431/13/209/prepub
